# High-throughput screening of nanoparticles in drug delivery

**DOI:** 10.1063/5.0057204

**Published:** 2021-08-26

**Authors:** Inês Tomé, Vitor Francisco, Hugo Fernandes, Lino Ferreira

**Affiliations:** 1 Biomaterials and Stem-Cell Based Therapeutics Group, Centre of Neuroscience and Cell Biology, University of Coimbra, 3060-197 Cantanhede, Portugal; 2 Faculty of Pharmacy, University of Coimbra, 3000-548 Coimbra, Portugal; 3 Faculty of Medicine, University of Coimbra, 3000-370 Coimbra, Portugal

## Abstract

The use of pharmacologically active compounds to manage and treat diseases is of utmost relevance in clinical practice. It is well recognized that spatial-temporal control over the delivery of these biomolecules will greatly impact their pharmacokinetic profile and ultimately their therapeutic effect. Nanoparticles (NPs) prepared from different materials have been tested successfully in the clinic for the delivery of several biomolecules including non-coding RNAs (siRNA and miRNA) and mRNAs. Indeed, the recent success of mRNA vaccines is in part due to progress in the delivery systems (NP based) that have been developed for many years. In most cases, the identification of the best formulation was done by testing a small number of novel formulations or by modification of pre-existing ones. Unfortunately, this is a low throughput and time-consuming process that hinders the identification of formulations with the highest potential. Alternatively, high-throughput combinatorial design of NP libraries may allow the rapid identification of formulations with the required release and cell/tissue targeting profile for a given application. Combinatorial approaches offer several advantages over conventional methods since they allow the incorporation of multiple components with varied chemical properties into materials, such as polymers or lipid-like materials, that will subsequently form NPs by self-assembly or chemical conjugation processes. The current review highlights the impact of high-throughput in the development of more efficient drug delivery systems with enhanced targeting and release kinetics. It also describes the current challenges in this research area as well as future directions.

## I. INTRODUCTION

The use of nanotechnologies for the prevention, diagnosis, and treatment of diseases in medicine can have a major impact on human health. The design and development of nanoparticles (NPs) for drug delivery is an area of nanomedicine that has received great attention. NPs are used to (i) increase therapeutic efficacy, (ii) decrease the therapeutically effective dose, and/or (iii) reduce the risk of systemic side effects; and these can be achieved by improving the solubility, by controlling the passive and/or active targeting, or by endogenously and/or exogenously triggering release of the payload.^1,2^ Despite their therapeutic advantages and the promising results obtained in preclinical studies, the number of NP-based products used in the clinic is still limited. In 2016, there were 25 Food and Drug Administration (FDA) or European Medicines Agency (EMA) approved nanomedicines, and more than 45 NP formulations that were under evaluation in clinical trials.^3^ Some recent approvals include two lipid formulations for patients with acute myeloid leukemia,^4^ treatment of polyneuropathy caused by transthyretin amyloidosis,^5^ and an inorganic formulation for oncology applications.^6^ There are also two authorized vaccines based in nanoparticle formulations (Pfizer-BioNTech^7^ and Moderna^8^) containing mRNA to prevent COVID-19 disease. Moreover, preclinical studies and clinical trials with NPs have shown promising results in cancer treatment^9^ and combined delivery of therapeutic agents, such as small molecules, genes, and biorelevant molecules.^10^

To increase the impact of nanomedicine in the clinic, several experimental challenges need to be addressed.^11^ From a biological perspective, the fate of intact and disassembled NPs *in vivo*, particularly their interaction with blood components and intercellular compartments, is critical to understand their mechanisms of action.^12^ From a nanomaterial perspective, parameters such as batch-to-batch reproducibility and scale-up production under GMP-compliance need to be addressed. In addition, the identification of nanomaterials able to target specific tissues and cells, and deliver intracellularly different types of biomolecules, are required. In most studies, the identification of the best formulation was done by testing a small number of formulations or by modification of pre-existent ones. Unfortunately, this is a low throughput and time-consuming process that likely prevents the identification of formulations with the highest potential and/or with a tailor-made composition for a given application. For example, the recent development of a siRNA delivery formulation for the treatment of hereditary transthyretin amyloidosis took more than 10 years of development.^13^ More than 300 ionizable lipids were synthesized to find the best formulation for the delivery of the siRNA in the liver of mice.^14^ High-throughput combinatorial design and screening of NP libraries may be an alternative to allow the rapid screening of multiple formulations to identify the ones with a specific release profile and/or with cell/tissue targeting specificity. Combinatorial approaches offer several advantages over conventional methods since they allow for the incorporation of multiple components with varied physical-chemical properties into a polymer that will subsequently form NPs by self-assembly or chemical conjugation processes.

In the last few years, several reviews have covered the advances in NP formulations for the delivery of biomolecules including non-coding RNAs and mRNAs.^15–18^ This review focus in the role of high-throughput synthesis of drug delivery systems may facilitate the translation of non-coding RNA and mRNA into the clinic. Therefore, this review discusses the current challenges and future directions of high-throughput screening for the development of more efficient drug delivery systems, particularly in the context of non-coding RNAs and mRNAs. Initially, we cover historic facts about HTS, how/when the HTS was applied to the area of drug delivery, preparation of NP libraries, and the impact of HTS approaches in medicine. Finally, we discuss how this research area might advance in the near future and the expected impact.

## II. HISTORIC FACTS ABOUT HIGH-THROUGHPUT SCREENING (HTS) OF DRUG DELIVERY SYSTEMS

HTS emerged as a valuable technique for new lead discovery, both for the academia and industry. HTS allows researchers to test, in a time- and cost-efficient manner, large libraries of compounds using miniaturized *in vitro* assays designed to interrogate a given biological function. HTS foundations can be traced to the pharmaceutical industry in the early 1990s and were driven by the: (1) urgency to outpace the drug developing pipeline of competitors, (2) advances in cell biology and genomics (Human genome project) that endowed companies with novel tools and know-how (e.g., synthesis of recombinant proteins and gene editing tools), and (3) investment in laboratory automation.^19,20^ Prior to the debut of HTS, screening of new drugs was a slow and costly process limited to around 100 000 candidates (∼20 000/year) with candidate molecules synthesized and kept dry on vials and weighted and diluted manually.^19^ Nowadays, the success rate of HTS in drug discovery has been reported to be approximately 50%.^21^ The libraries of most big pharma companies engaged in HTS are between 0.5 and 4 × 10^6^ compounds.^22^ In some cases, the hits need to be further optimized to yield a drug of interest. From 58 drugs (for which the starting lead was known) approved between 1991 and 2008, 19 drugs have been identified by HTS.^21^ HTS assays were designed to be safe, sensitive, robust, and cost-effective. Typically, an HTS assay required the preparation and optimization of a library of compounds, followed by a primary screening where the readout was suitable to interrogate a large number of compounds in a fast and cost-efficient manner. Upon identification of “hits,” confirmatory screenings were designed and implemented, and the identified “leads” were tested in more complex *in vitro* and *in vivo* models prior to clinical trials.

**FIG. 1.**
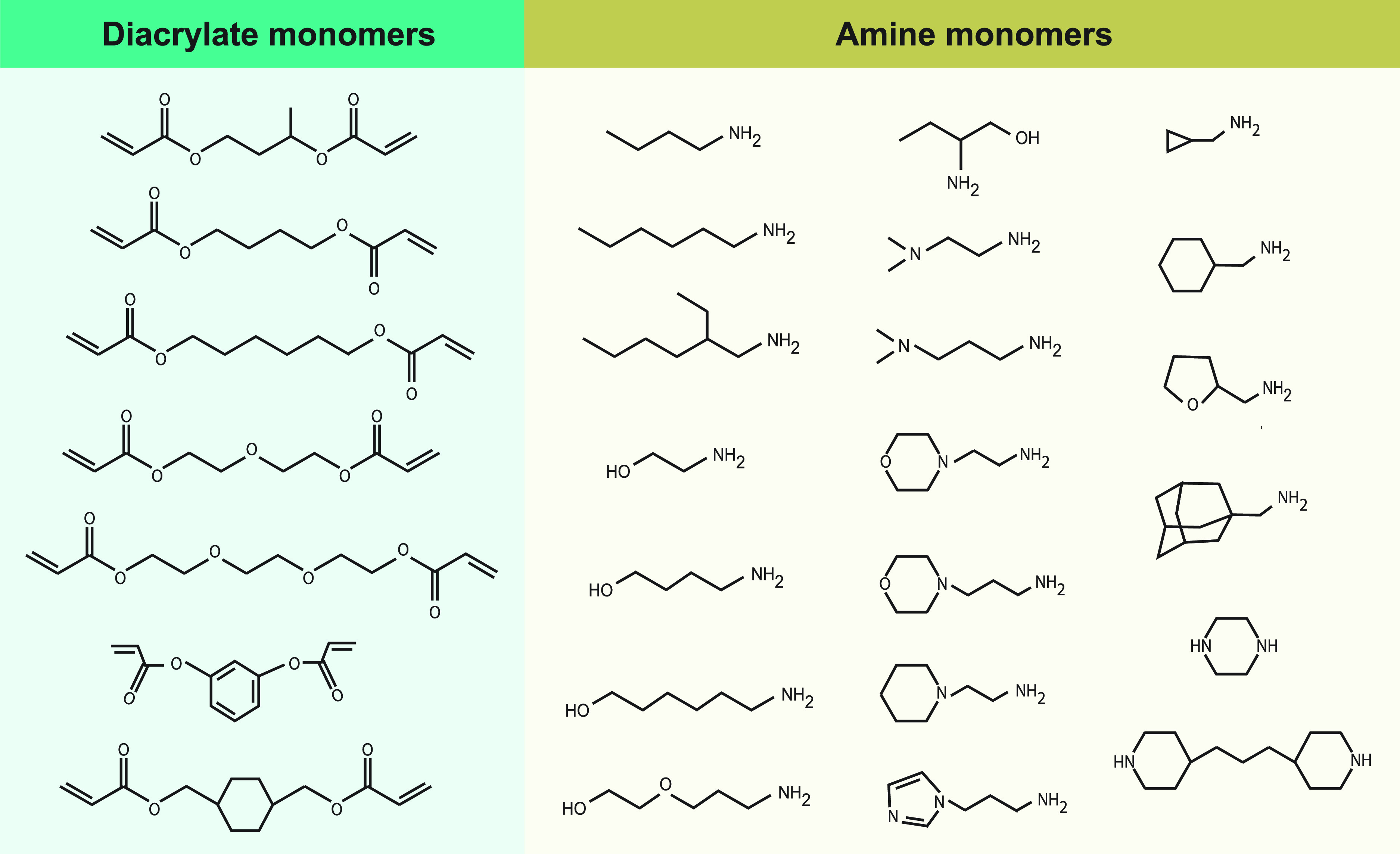
Semi-automated synthesis of a large library of degradable cationic polymers. The synthesis of the initial library of poly(β-amino ester)s polymers followed a combination of 7 diacrylate and 20 amine monomers, which resulted in 140 unique structures. This breakthrough work opened a new path in the field of nanoformulations for controlled drug delivery and was crucial for the development of automated HTS synthesis and characterization of polymeric libraries that succeeded. Reprinted with permission from Lynn *et al.*, J. Am. Chem. Soc. **123**, 8155–8156. Copyright 2001 American Chemical Society.^23^

The introduction of HTS to the field of nanomaterials for controlled drug release has been pioneered by Langer and co-workers in the beginning of the 21st century^23,24^ (Table I). Initial focus was given to polymeric libraries, because the starting materials were easily available and cheap, and the synthesis process was done without intermediary purification steps. The studies reported the synthesis and unbiased screening of a large library of biodegradable cationic polymers and oligomers for their capacity to work as gene delivery systems (Fig. 1).^25^ These initial steps were then followed by HTS studies with libraries of linear-dendritic hybrid polymers (that assemble with DNA to form NPs),^26^ libraries with photodegradable photocrosslinked materials,^27^ and lipid-like nanoformulations for DNA, siRNA, and mRNA delivery, both in *in vitro* and *in vivo* models.^28–31^ In many of these studies, the HTS comprised of the high-throughput synthesis of large NP libraries followed by their testing in cell models and finally validation in an *in vivo* model. More recently, and because of the poor correlation between *in vitro* and *in vivo* potency of the formulations, researchers have moved directly to *in vivo* models after NP synthesis.^32^

**TABLE I.** Examples of combinatorial libraries of NPs.

Year	Library size	Method	Chemistry	Selected references
**Cationic polymers and oligomers**				
	First generation libraries		Michael-type addition (conjugation addition of primary or secondary amines to diacrylates)	
2001	140 polymers	Low throughput		23 and 24
2003	2350 polymers	Semi-automated		69
2003	24 polymers	HTS		47
	Second generation libraries:			
2005	>500 polymers	HTS		48–50
			
2005	486 polymers	HTS (reduction to milligrams scale of the original library)		51–54
			
2007	Third generation libraries:			55, 81, and 104
	Photopolymerized	HTS (polymeric microarrays with robotic fluid handling)	Michael-type addition, followed by polymerization of amines and acrylates with a light-activated radical initiator	
2004	576 polymers			112
2005	1152 polymers			113
2005	1700 polymers			114
2006	120 polymers			27
	Terpolymers	HTS	Polymerization of diacrylates, a hydrophobic alkylamines and hydrophilic amines Ring opening polymerization and Michael step‐growth polymerization	
2013	80 terpolymers			87
2016	6 terpolymers			88
2018	16 terpolymers			89
Lipid-polymer hybrids				
2014	500 NPs	HTS	Epoxide ring-opening reaction (conjugation of epoxide-terminated lipids to low MW polyamines)	115
	Extended work			
	Polymer 7C1			57–59
Lipid-like nanoparticles				
	First generation libraries:	HTS	Michael-type addition Epoxide ring-opening reaction (by amine substrates)	
2008	1200 NPs (pilot library: 700 NPs)			28, 30, and 31
		
2010	126 NPs			56, 60, and 61
	Second generation libraries:	HTS	Michael-type addition One-pot synthesis (thiolactone ring opening)	
2010	51 NPs			73
2011	3780 NPs			74
2012	54 NPs			29
2013	32 LNPs			116
		
2014	1400 NPs			43
2018	288 NPs			67 and 102
	Microfluidics	HTS		
2012	70 NPs			117
2020	14 NPs			90

## III. HIGH-THROUGHPUT SYNTHESIS AND SCREENING OF NPS

**FIG. 2.**
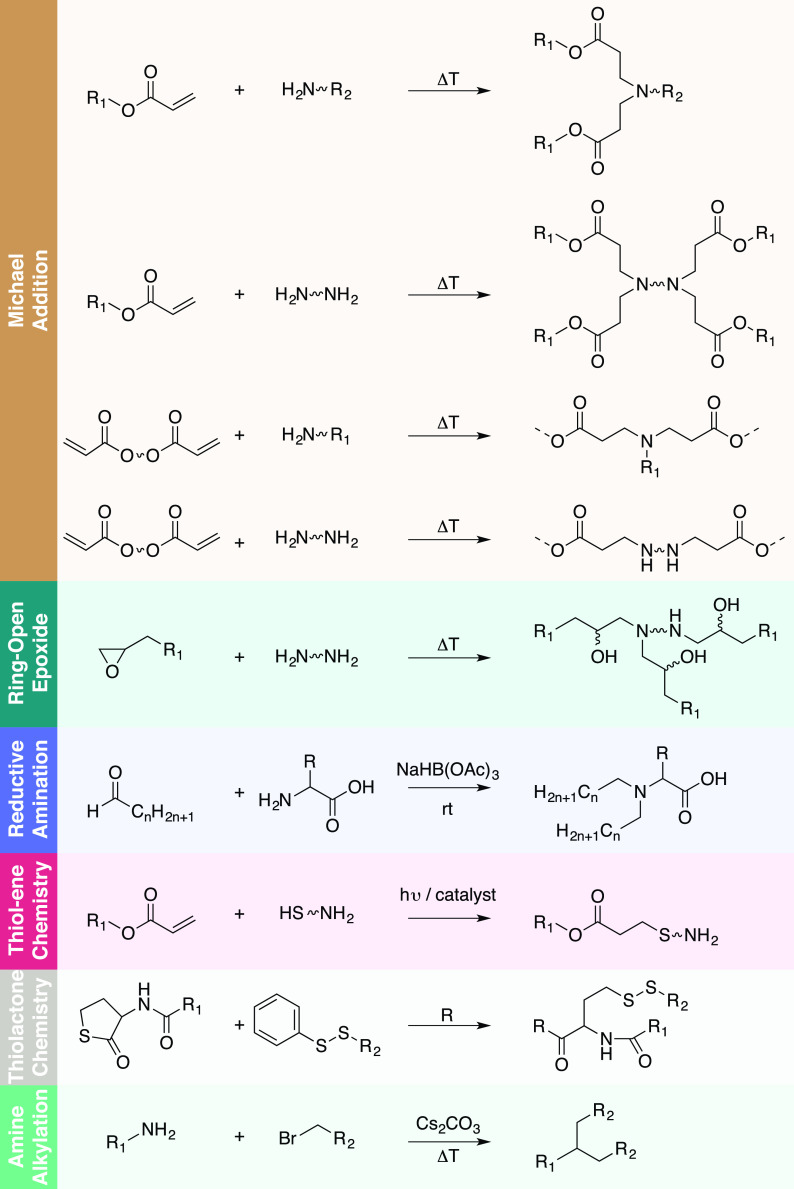
Chemical reactions used for the combinatorial synthesis of polymers and lipid-like materials to create nanoparticle libraries.

Combinatorial approaches for the synthesis of polymers or lipids offer several advantages over conventional methods, since they allow the integration of multiple components with varied properties into a nanosystem via self-assembly or chemical conjugation. Because of the number of studies, we will mostly focus on polymeric NPs, but, in general, the approaches can be extrapolated for the synthesis of lipid NPs. High-throughput synthesis and screening is crucial in polymer design, because polymer composition directly affects properties such as drug loading, stability in circulation, and targeting to different cell types. The first approach relies on the self-assembly of macromolecular building blocks with specific functionalities to yield a large variety of NP systems. These self-assembled nanosystems with diverse functionalities can then be rapidly screened in a high-throughput fashion for selection of the best candidates, or hits, which are further evaluated for safety and efficacy. Parallel synthesis of combinatorial polymeric libraries can be achieved manually or robotically both in solid support and in solution. In solid-phase assisted synthesis, sequence-defined polymers can be prepared with high precision using several synthetic strategies such as phosphoramidite coupling, thiolactone/Michael, triazine, thiol-ene, and CuAAC click chemistry.^33,34^ For example, artificial oligoamino acids, such as succinoyl-tetraethylene pentaamine and succinoyl-pentaethylene hexamine, in combination with natural α-amino acids, have been used to generate sequence-defined cationic polymers.^35^ Using this strategy and a step-by-step optimization of topology,^36^ inclusion of small chemical delivery motifs such as tyrosine trimers,^37^ histidines,^38^ fatty acids,^39^ disulfide-forming groups,^40^ and targeting ligands,^41,42^ it was possible to synthesize a large library of more than 1000 oligomers. Despite the advantages of using solid-phase synthesis of polymers, several disadvantages can be associated with this methodology such as (i) limited scalability, (ii) excessive use of reagents, (iii) additional deprotection steps after coupling reaction, and (iv) need for very efficient coupling reactions. Alternatively, liquid synthesis uses well-established reaction strategies and easily scalable methods. Large chemically diverse libraries have been synthesized using simple synthetic routes including, but not limited to, Michael addition,^28,43–55^ epoxide chemistry,^56–61^ reductive amination,^62^ thiol-ene/thiol-yne chemistry,^63–66^ thiolactone chemistry,^67^ and direct alkylation of amines^68^ (Fig. 2). For example, Michael addition polymerizations have been extensively investigated in the synthesis of linear or hyperbranched poly(β-amino ester)s (PBAE) and poly(amido amine)s (PAA).^23^ The PBAEs and PAAs are synthesized via a one-pot Michael addition of amines to acrylates without production of any side products. A large library comprising 2350 polymers has been synthesized for gene delivery applications.^69^ A unique advantage of this method was that the synthesis of the polymers and their testing could be performed in “one-pot” without the need for purification or solvent removal, thus allowing the screening of large libraries involving a reduced number of steps. Michael addition was also used in the synthesis of several libraries of lipidoids mainly used for gene delivery.^28,70–74^ Nevertheless, despite its simplicity, this synthetic strategy requires high temperature or long reaction time (up to several days). To overcome this limitation, other synthetic reactions were explored. Typically, thiolactone and thiol/ene chemistries require less time for the synthesis of polymers. For example, a library of 840 polyesters has been synthesized based on a polycondensation reaction of trimethylolpropane allyl ether and diacyl chlorides during 24 h, followed by the functionalization with amino and alkyl amines by thiol-ene addition.^64^ Other libraries have been synthesized based on a one-pot three-component reaction via thiolactone opening by an amine, followed by a disulfide exchange reaction.^75^ The reaction to obtain the 25 lipidoids was completed after 2 h and was performed at room temperature. It should be noted that in certain cases, long synthetic procedures or complicated purification steps were major drawbacks in the synthesis of nanomaterials libraries.^72^ The elimination of steps in polymer purification, such as the use of rotary evaporator or precipitation in a solvent to obtain the purified polymer, removed a time-consuming step in the high-throughput process.^76^ A multitude of design parameters and combinations thereof can contribute to the precise fine-tuning of a highly efficient material for a specific application. It is worth mentioning that the biocompatibility and biodegradability of any synthetic polymer used to synthesize NPs for intravenous injection are highly strict. For a material, the biocompatibility refers to its ability to perform its function with an appropriate host response. This means that the material itself, as well as all the degradation products released during its lifetime, must not elicit any toxic effect in the host.^77^ In turn, the biodegradability of the material has to be ensured to avoid the accumulation of NPs in the body.

### A. Type of NP libraries: Compositional vs combinatorial

NP libraries are often divided into compositional or combinatorial libraries. Compositional libraries consist of NPs with different chemical structures (e.g., metal, metal oxides, silica, carbon, and polymers) but similar physicochemical properties.^78–81^ By combining numerous elements found in the periodic table, these libraries offer a vast palette of multi-element nanomaterials. On the other hand, combinatorial libraries contain NPs with the same chemical configuration but vary one major physicochemical property (e.g., size, shape, surface functionalization, charge, and chemistry). Most of the HTS studies reported so far have reported the use of combinatorial libraries.^44,82–89^ HTS and combinatorial design of NP libraries can be a valuable approach to allow rapid and simultaneous screening of multiple formulations with specific release and cell/tissue targeting properties. For example, a combinatorial library of lipid NPs was developed for the *in vivo* delivery of siRNAs to leukocytes.^90^ These amino lipid molecules were constructed using a microfluidic system that assembled cholesterol, ionizable lipid, 1,2-distearoyl-sn-glycero-3-phosphocholine (DSPC), and PEGylated lipids. Modifications to the linker backbone, lipid chain, and/or the headgroups resulted NPs with different sizes that ultimately led to differences in cell viability, transfection efficiency, internalization, biodistribution in organs, and ultimately differences in gene silencing efficiency in leukocytes. NPs with a hydroxylamine linker backbone had improved gene silencing in lymphocytes, while NPs with a hydrazine linker had no effect. Thus, the importance of using diverse libraries of NPs is crucial to identify formulations capable of eliciting the desired biological response while overcoming deleterious side-effects. The selection of the starting material should be the first step on the development of an HTS assay and should take into consideration downstream applications such as the route of administration of the nanoformulation or the potential cytotoxic effects from the nanomaterials.

### B. Assay design

Development and screening of nanomaterial libraries with different compositions and properties will contribute to accelerate the identification of the most suitable delivery system for a given biological application. Yet, developing suitable screening platforms with high accuracy, coupled with a rapid method of analysis, is challenging. In many cases, the selection of lead compounds relies on an extensive characterization of the NP library using cellular models for the *in vitro* screening followed by the selection of a limited number of formulations for *in vivo* testing.^91–96^ Yet this paradigm is changing since recent studies have adopted to test the NP formulations after their synthesis immediately in animal models.^14,97^

A well-designed and implemented cell-based HTS assay can provide several outputs such as NPs toxicity, uptake, and efficacy data. In the HTS performed in the last 20 years, the following cellular readouts have been reported: (i) gene knockdown,^91,92^ (ii) gene knockout,^93,94^ (iii) cell viability,^98,99^ and (iv) expression of a cellular reporter.^95,96^ In most of these HTS studies, the effect of the nanoformulations was evaluated by optical methods including absorbance,^100^ fluorescence,^101^ and luminescence,^28,56^ using plate readers or high content microscopes.^102,103^

**FIG. 3.**
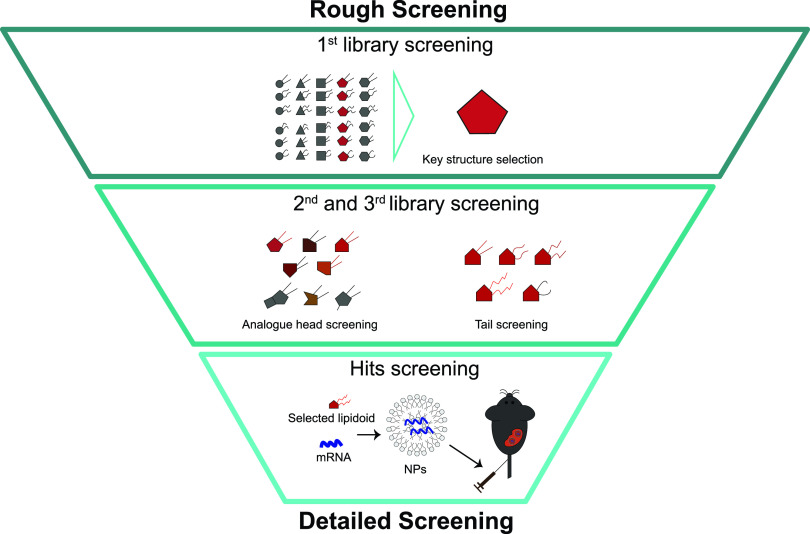
A systematic representation method of a lipidoid library of compounds for the mRNA delivery to T lymphocytes from a rough-to-detailed screening. Reproduced with permission from Zhao *et al.*, Angew. Chem. Int. Ed. **59**, 45 (2021). Copyright 2020 John Wiley and Sons.^72^

Drug development usually follows an inverted pyramid approach to organize and generate knowledge (Fig. 3). Usually, following the primary screening, multiple confirmatory screenings are performed to validate the original hits. While in the first screening, a rough approach is conducted to identify selective and efficient compounds, during secondary screenings, a more detailed analysis can be run to further validate compounds using different types of assays. Secondary assays may rely on the use of (i) purified NPs, (ii) NPs tested at different doses, (iii) in different types of cells, (iv) synthesis of NPs in large scale, among others. Secondary screens can confirm the hits of the primary screen and provide insights regarding the mechanism of action. As an example, a secondary screen was important to show the effect of polymer molecular weight (MW) in the bioactivity of the nanoformulations.^104^ Two polymers with identical structures but with different MWs had distinct cell activity. Secondary screens have also been performed to demonstrate the intracellular delivery efficiency and cellular tropism of hit formulations. For example, the uptake of dual-ligand liposomes was initially demonstrated in a 2D cell culture model, followed by 3D tumor spheroid models and finally by *in vivo* studies.^105^

### C. Selection of candidates and *in vivo* testing

Following an extensive characterization of the NP library using *in vitro* models, one should be able to select a small group of NPs for *in vivo* evaluation. To overcome the lack of information pertaining, for example, the toxicity of the NPs at the organism-level, the use of small well-characterized organisms for high-throughput toxicological assessment emerged as an important strategy. Among these, *Caenorhabditis elegans*^106^ and *Danio rerio* (zebrafish) models^107,108^ have been used in a high-throughput manner to assess NP's toxicity. Notwithstanding, complex mammalian organisms, such as rodents and non-human primates, are important at the preclinical stage to obtain pharmacological information data due to their similarity with humans.^62,109^ Recently, advances have been made to predict, using high-throughput *in vitro* assays, the *in vivo* behavior of NPs. For example, a chemically diverse library of 30 lipid NPs was constructed in order to perform a fast screening of NPs biodistribution in tissues and cells.^110^ Each of these NPs carried particular nucleic acid cargos—termed “barcodes”—that were administered as a single pool to mice. Using deep sequencing as a high-throughput methodology, the authors were able to identify the exact location of these NPs in the mice lungs, heart, or liver with high sensitivity and specificity. This target-specific approach overcomes the off-target delivery of NPs that often occur *in vitro*, hasten up the pre-clinical studies and contributes to the study of the relation of chemical structure and biological function. Later, the authors also demonstrated that this barcode system could be used to track more than 150 NPs, at the same time, *in vivo.*^111^ However, one of the limitations of this methodology lies on the fact that only NPs that are stable prior to parenteral administration will be retained *in vivo*, thus missing potentially interesting nanoformulations. Importantly, this barcode strategy was used to demonstrate that the *in vitro* delivery of NPs in static cell cultures did not predict the *in vivo* delivery, one of the major drawbacks of the use of nanomaterials.^111^

## IV. IMPACT OF HTS IN DRUG DELIVERY

HTS has an important role in the progress of the drug delivery area, because it: (i) allows the identification of formulations without the need of an hypothesis and (ii) allows structure-function relationship analyses, providing new insights that can be used to assist design and optimization of new libraries.^112–114^ The use of HTS allowed researchers to understand the impact of factors such as the polymer's MW,^115^ the pKa,^43,116^ the hydrophilicity/hydrophobicity of the hydrocarbon chain,^43,81,117^ the type of amine groups,^43,118^ type of end-functional groups,^81,104^ the degradation kinetics,^91,92,119^ and the number and saturation level of the hydrocarbon chains.^102^ For example, from a library of 2000 NPs, the most effective NP formulation for siRNA delivery was formed by a low MW polymer.^115^ In addition, HTS studies in 32^116^ or 1400^43^ lipid formulations showed that NPs pKa has a strong correlation with *in vitro* and *in vivo* data than NPs size or NPs loading. Indeed, HTS studies showed that lipid nanoformulations with a pKa in the range of 5.5–7 had higher *in vitro* an *in vivo* activity than the ones with low pKa.^43,116^ Moreover, other HTS studies indicate that parameters, such as NP disassembly and NP internalization, do not correlate with formulation activity.^91,92,119^

HTS was important for early steps in the delivery programs of non-coding RNAs.^28^ The development of Patisiran, a lipid NP containing siRNA for the treatment of hereditary transthyretin amyloidosis,^120^ required more than 10 years of development and the screen of more than 300 ionizable lipids.^14^ The approved NP formulation contains the ionizable cationic lipid Dlin-MC3-DMA, which has a head group containing tertiary amines that are uncharged at neutral *p*H but protonated under acidic conditions, helper lipids, and PEG-containing lipids.^121^ Many aspects of the NP formulation have been carefully designed such as the size, encapsulation efficiency, scalable manufacturing processes, and formulation stability. Yet the delivery of biomolecules to other organs than liver remains a challenge. Recent progresses with lipid NPs seem to indicate that the incorporation of specific charged lipids in lipid NPs may control the tropism of the NPs to specific organs (liver, lung and spleen).^97^ Yet, more progresses are needed to develop NP formulations able to reach with high efficiency organs such as the heart or the brain.

**FIG. 4.**
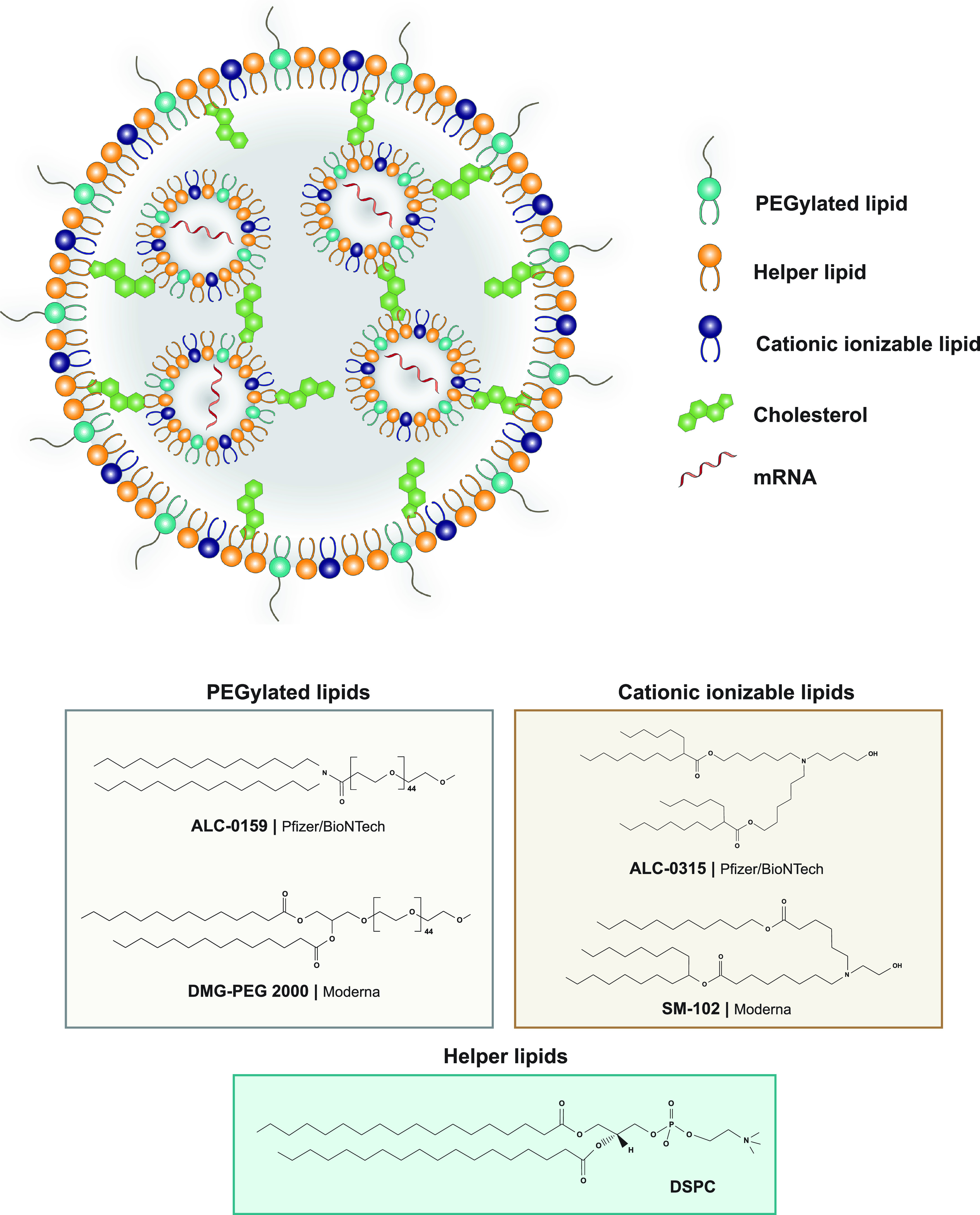
Lipid NPs technology for the delivery of SARS-CoV-2-based mRNA. The delivery of mRNA requires a delivery system that protects the nucleic acids to be degraded by RNAses or the immune cells, because they are negatively charged and have large size. Both Pfizer/BioNTech (BNT162b2) and Moderna (mRNA1273) nanomedicine vaccines have similar compositions: (1) ionizable cationic lipids, that when in low *p*H are positively charged, thus allowing the complexation with nucleic acids, while at physiological pH have neutral charge, reducing possible side effects; (2) PEGylated lipids, that reduce opsonization and clearance; (3) helper lipids, that promote cell binding; (4) cholesterol, to mechanically stabilize the nanoparticle by occupying the gaps between the lipids. ALC-0159, 2-[(polyethylene glycol)-2000]-N,N-ditetradecylacetamide; DMG-PEG 2000, 1,2-dimyristoyl-*rac*-glycero-3-methoxypolyethylene glycol-2000; ALC-0315 ((4-hydroxybutyl)azanediyl) bis(hexane-6,1-diyl)bis(2-hexyldecanoate); SM-102, heptadecan-9-yl 8-((2-hydroxyethyl)(6-oxo-6-(undecyloxy)hexyl)amino)octanoate; DSPC, phospholipid distearoylphosphatidylcholine.

The information collected from HTS studies was critical for the rapid development of COVID-19 mRNA vaccines.^7,8^ The mRNA of some COVID-19 vaccines is released by lipid NPs (Fig. 4). These NPs have four components: ionizable lipids to allow the interaction with mRNA, pegylated lipids to increase circulation time of the formulation by preventing opsonization of plasma proteins and the uptake of macrophages, and phospholipids and cholesterol that contribute for the NP structure. These NP formulations may be further improved to protect the mRNA overtime, potentially without requiring so low temperatures of storage, and potentially to increase their intracellular efficacy.

## V. FUTURE PERSPECTIVES

In this review, we have covered recent progresses in the synthesis of combinatorial nanomaterials for the delivery of biomolecules. The use of simple, convenient, and efficient chemical synthesis strategies has facilitated the parallel and combinatorial synthesis of small and large libraries of nanomaterials with diversified chemical structures. Indeed, chemistry has a crucial role in accelerating material discovery, and this will lead to new types of nanomaterials with different physical-chemical properties capable of increasing targeting and responsiveness.

We anticipate several progresses in the area of HTS applied to drug delivery by the: (i) use of *in vitro* models of increasing complexity (e.g., spheroids, micro-tissues, and organoids), (ii) use of techniques with single cell resolution, (iii) combinatorial screening by automated microfluidics, (iv) use of better structure-activity correlation models, and (v) more effective development of NP formulations able to target organs other than liver.

Regarding the first point, in many diseases, the inhibition of a single target is not enough to have a therapeutic proposition. In addition, cells do not behave in the same way when are cultured in monoculture or in a living tissue. Therefore, a more faithful *in vitro* representation of the *in vivo* biology will impact HTS in the development of more efficient drug delivery systems. It is important to mimic, in as much as possible, the *in vivo* cellular environment by controlling, for example, the flow, type of extracellular matrix, intercellular communication (particularly with vascular and immune cells), cellular heterogeneity, or even the off-target effect of the NPs. The *in vivo* complexity can be achieved by using co-cultures systems, 3D culture platforms, patient-derived adult- or iPSC-derived cells, and even microfluidic “organ-on-a-chip” models.^98,122,123^ In the last 30 years, some studies have used three-dimensional (3D) cell cultures in order to recapitulate the complexity of the *in vivo* organization. These 3D cell cultures can adopt different formats such as multicellular structures derived from more than one type of cell (e.g., micro-tissues and co-culture systems), cell aggregates from a single cell type (also termed spheroids),^124^ and organoids that are derived from cells differentiated from a progenitor cell population with capacity to form organ-like structures.^125^ Many of HTS studies documented so far with nanomaterial libraries have not explored the use of 3D cell cultures or intact organs *ex vivo*.^126^ Aside from recapitulating the *in vivo* environment or pathophysiological conditions, these are important for understanding the NPs trafficking, targeting, and biological effects at the subcellular level. The use of whole organs requires the use of appropriate tissue-clearing methods coupled with complex imaging systems and algorithms to observe the complex tissue architecture.^127^ This is an area that will grow in the coming years, as it is now being explored for drug discovery and precision medicine.^128^ However, there are major limitations of 3D cell culture that still need to be overcome, namely, of: (i) scalability to multi-well microplates, (ii) automation, (iii) need of appropriate imaging systems for the visualization of 3D structures in an high throughput fashion (e.g., automated high-throughput light-sheet fluorescence microscopy that was only launched in 2018),^129^ (iv) compatibility of systems (from NP library synthesis and liquid handling equipment to automated screening and analysis), and (v) reproducibility.

Regarding the second point, the readouts of most HTS studies are limited to major alterations in cell phenotype such as morphology, proliferation, migration, and cell death. It is expectable that in the near future, the readouts in HTS will increase in complexity by the use of single-cell transcriptome sequencing (scRNA-seq)^130^ as well as single cell proteomics.^131^ Recent advances in these techniques enable cost-effective HTS at single cell resolution. For example, the cost of scRNA-seq library preparation is now less than $0.01 *per* cell allowing the profiling of millions of cells *per* experiment.^132^ This has been demonstrated recently in the screen of 188 compounds in three cancer cell lines. The authors have profiled approximately 650 000 single cell transcriptomics across 5000 independent samples in one experiment. The results highlighted the heterogeneity in cellular response to a perturbation induced by a chemical compound and the possibility to distinguish the effect of a unique compound in different cell populations in a tissue or a 3D *in vitro* model.

Regarding the third point, the HTS studies performed so far are unable to provide combinatorial and dynamic drug treatments. Microfluidics offers a simple to use, rapid, low-cost (with low reagent consumption), and high-throughput approach for the development of NPs' libraries with controlled structures that increase the reproducibility of assays.^117,133^ Even though microfluidics had a great evolution in the synthesis of NP libraries, its use on combinatorial screening is far from sufficient. Recent advances in microfluidic systems enable the performance of multiple assays in parallel in highly reproducible environment with the utilization of minimal volumes of cell culture media as well as formulations.^134^ These automated systems may open other avenues of research in the area of HTS for drug delivery systems, because they allow the alteration of the concentration of the formulation as well as the timing and derivation of their effect in 2D or 3D cell cultures.

Regarding the fourth point, recent advances in machine learning and the capacity to model big data may allow us to infer better structure: function relationships.^135,136^ For example, the phase behavior of lipid NPs has been successfully predicted using machine learning. By varying ratios of saturated and unsaturated fatty acids or the chain length of the lipids, it elucidated the contributions of various factors and may serve as a bridge to deduce the delivery efficiency of the formulations in *in vitro* or *in vivo*.^137–139^ In another example, the integration of high-throughput experimentation with machine learning led to the identification of 100 drug NPs with high loading capacity.^140^ In addition, the use of machine learning tools can be envisioned as a strategy to identify new principles for more efficient release^141^ and targeting^135^ to specific cells.

Regarding the fifth point, the translation of mRNA and gene editing therapies require the development of delivery systems with tropism to specific organs. Current NP formulations have poor targeting efficiency to organs beyond the liver. A recent study has reported the development of lipid NPs to target organs such as the liver, lung, or spleen following NP intravenous administration.^97^ In this case, the incorporation of a charged lipid component in the NP formulation affected their organ targeting capacity. Yet, further progresses are needed to develop NP formulations able to target efficiently organs such as the heart or the brain. Therefore, in the coming years, it is expectable that HTS of NP formulations with variable effect of morphology, charge, pKa, hydrophilicity/hydrophobicity, among other factors, will be developed to address this issue.

## VI. CONCLUSION

In summary, high-throughput combinatorial synthesis and screening have proven to be a powerful strategy to obtain efficient nanoparticles for drug delivery with clinical relevance, particularly non-coding RNAs and mRNAs. It is expectable that in coming years, the combination of HTS of NP formulations with organoids and the use of scRNA-seq and machine learning tools to determine the biological impact of the released drugs will have a significant impact in the design of more efficient drug delivery systems.

## Data Availability

Data sharing is not applicable to this article as no new data were created or analyzed in this study.
